# Crystal structure of *Pelagibacterium halotolerans* PE8: New insight into its substrate-binding pattern

**DOI:** 10.1038/s41598-017-04550-7

**Published:** 2017-06-30

**Authors:** Ying-Yi Huo, Suhua Li, Jing Huang, Zhen Rong, Zhao Wang, Zhengyang Li, Rui Ji, Siyun Kuang, Heng-Lin Cui, Jixi Li, Xue-Wei Xu

**Affiliations:** 1 Key Laboratory of Marine Ecosystem and Biogeochemistry, Second Institute of Oceanography, State Oceanic Administration, Hangzhou, 310012 China; 2 State Key Laboratory of Genetic Engineering, Collaborative Innovation Center of Genetics and Development, School of Life Sciences, Shanghai Engineering Research Center of Industrial Microorganisms, Fudan University, Shanghai, 200438 China; 3 College of Food and Biological Engineering, Jiangsu University, Zhenjiang, 212013 China

## Abstract

Lysophospholipase_carboxylesterase (LPCE) has highly conserved homologs in many diverse species ranging from bacteria to humans, as well as substantial biological significance and potential therapeutic implications. However, its biological function and catalytic mechanism remain minimally investigated because of the lack of structural information. Here, we report the crystal structure of a bacterial esterase PE8 belonging to the LPCE family. The crystal structure of PE8 was solved with a high resolution of 1.66 Å. Compared with other homologs in the family, significant differences were observed in the amino acid sequence, three-dimensional structure, and substrate-binding pattern. Residue Arg79 undergoes configuration switching when binding to the substrate and forms a unique wall, leading to a relatively closed cavity in the substrate-binding pocket compared with the relatively more open and longer clefts in other homologs. Moreover, the mutant Met122Ala showed much stronger substrate affinity and higher catalytic efficiency because less steric repulsion acted on the substrates. Taken together, these results showed that, in PE8, Arg79 and Met122 play important roles in substrate binding and the binding pocket shaping, respectively. Our study provides new insight into the catalytic mechanism of LPCE, which may facilitate the development of structure-based therapeutics and other biocatalytic applications.

## Introduction

Esterases have important physiological roles and biotechnological applications because they can catalyze the hydrolysis of short-chain ester-containing molecules and produce carboxylates and alcohols^1–3^. Esterases belong to the lysophospholipase_carboxylesterase family (the LPCE family)^4^ and were previously classified in bacterial family VI by Arpigny and Jaeger^1^. This family includes the smallest carboxylesterase (23–26 kDa) found to date, and bacterial carboxylesterases show high sequence similaritiy with their eukaryotic counterparts (~40%)^1^. LPCE family proteins play significant roles in human diseases. For example, human putative Gα-regulatory protein acyl thioesterase (APT1) has been well characterized as a modulator in the Ras signaling pathway and has been confirmed as a target for cancer therapeutics^5, 6^. Human lysophospholipase-like 1 (LYPLAL1) might be a triacylglycerol lipase involved in obesity^7, 8^. In addition, a bacterial carboxylesterase (FTT258) from *Francisella tularensis*, a causative agent of tularemia^9, 10^, has been investigated as a novel drug target^11^. Overall, LPCE family enzymes are increasingly pharmaceutically interesting as potential therapeutic targets. Nevertheless, our understanding of the LPCE family is very limited. Currently, the crystal structures of only six LPCE family proteins have been reported, including *Rhodobacter sphaeroides* RspE^12^, *Pseudomonas aeruginosa* PA3859^13^, *P*. *fluorescens* esterase II^14^, *F*. *tularensis* FTT258^11^, human APT1^15^ and human LYPLAL1^7^.

The LPCE family member PE8 was recently identified by our group^16, 17^. The biochemical characterization of PE8 revealed that it is an alkaline esterase and a potential industrial biocatalyst^18^. PE8 exhibits enantioselective hydrolysis of prochiral dimethyl 3-(4-fluorophenyl)glutarate (3-DFG), generating (*R*)-3-(4-fluorophenyl)glutarate ((*R*)-3-MFG)^18^, a precursor of important pharmaceutical compounds, such as the antidepressant (−)-paroxetine hydrochloride^19, 20^. In this study, we obtained and analyzed the crystal structure of PE8 to gain new insight into the catalytic mechanism of LPCE family enzymes.

## Results and Discussion

### Overall structure

**Figure 1:**
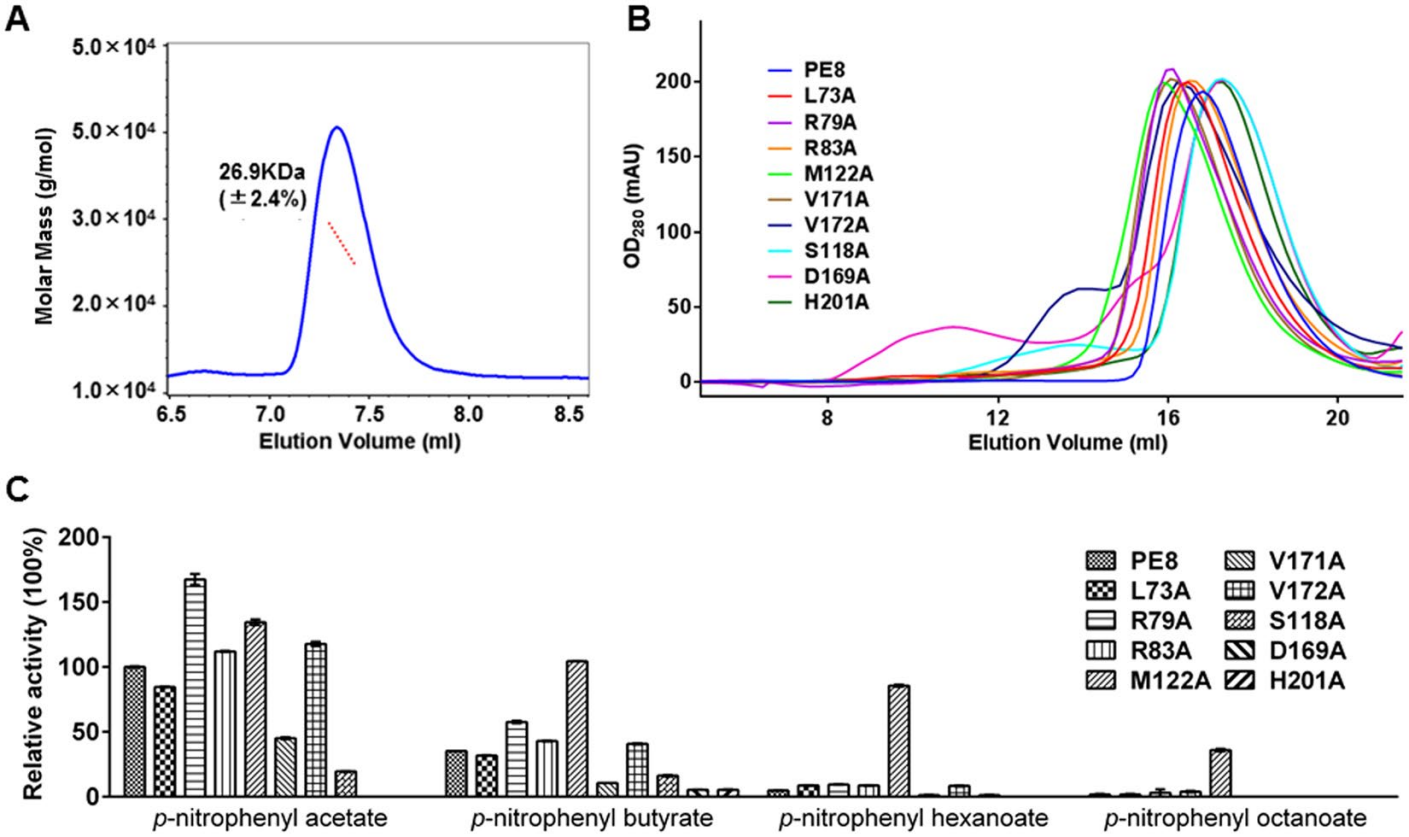
Gel filtration profile and enzymatic activities of PE8 and mutants. (**A**) MALS analysis of PE8. The MW of PE8 was 26.9 kDa (±2.4%), indicating that PE8 is a monomer in solution. (**B**) Gel filtration profiles of PE8 and mutants on a Superdex 200 10/300 column. Wild-type PE8 and its mutants formed monomers in solution. Mutants L73A, R79A, R83A, M122A, V171A and H201A changed slightly, whereas mutants V172A, S118A and D169A showed heterogeneity upon gel filtration. (**C**) The enzymatic activities of wild-type PE8 and its mutants were determined using the following substrates: *p*-NP acetate, *p*-NP butyrate, *p*-NP hexanoate and *p*-NP octanoate.

The diffracting dataset of the PE8 crystal was integrated into monoclinic space group P2_1_ with two molecules per asymmetric unit and a resolution of 1.66 Å. However, multi-angle light scattering (MALS) analysis showed that the molecular weight (MW) of PE8 was 26.9 kDa (±2.4%) (Fig. 1A), consistent with the theoretical MW of 6× His fusion PE8 (25.4 kDa), and revealed that PE8 existed as a monomer in solution. Additionally, 329 water molecules, one polyethylene glycol (PEG) monomethyl ether (MME) 550 molecule and one glycerol molecule were modeled. The final refined model had an *R*_work_ of 16.84% and an *R*_free_ of 20.52%. The crystallographic statistics for data collection and structure refinement are summarized in Table 1.

**Table 1:** Statistics from the data collection and refinement of PE8.

Parameters	PE8
**Diffraction data**	
Wavelength (Å)	0.9785
Resolution range (Å)	50.0–1.66 (1.69–1.66)^a^
Space group	P 2_1_
Unit cell	
a, b, c (Å)	41.772, 73.398, 66.403
α, β, γ (°)	90.00, 102.37, 90.00
Unique reflections	46061
Completeness (%)	99.1 (99.6)
*R*_merge_ (%)^b^	8.4 (47.1)
I/σ (I)	22.5 (3.17)
Wilson B-factor	21.42
**Refinement statistics**	
Resolution range (Å)	36.7–1.66 (1.72–1.66)^a^
*R*_work_^c^/*R*_free_ (%)^d^	16.84/20.52
No. atoms	3580
No. residues	438
No. PEG MME 550	1
No. glycerol	1
No. water molecules	329
**RSMD**	
Bond lengths (Å)	0.020
Bond angles (°)	2.02
Average B-factor (Å^2^)	26.37
Ramachandran favored (%)	98
Ramachandran outliers (%)	0.46
**PDB code**	5DWD

^a^Values in parentheses refer to data in the highest resolution shell. ^b^ *R*_merge_ = ∑|I_i_ − 〈I〉 |/∑|I|, where I_i_ is the intensity of an individual reflection and is the average intensity of that reflection. ^c^ *R*_work_ = ∑||F_o_| − |F_c_||/∑|F_o_|, where F_o_ and F_c_ are the observed and calculated structure factors of reflections, respectively. ^d^ *R*_free_ was calculated as *R*_work_ using 5% of the reflections that were selected randomly and omitted from refinement.

**Figure 2:**
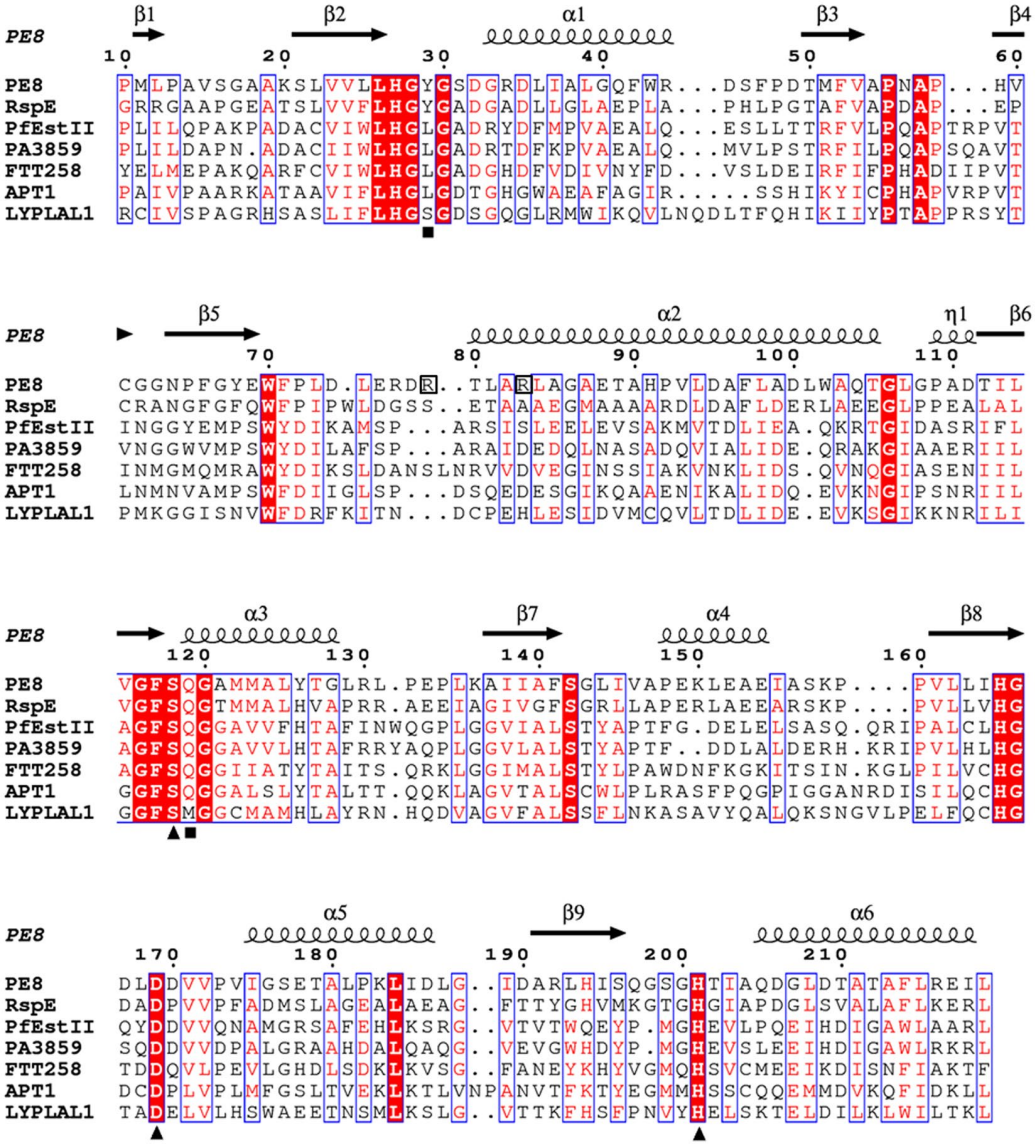
Amino acid sequence alignment of PE8 (PDB: 5DWD) with homologs from the LPCE family. RspE, from *R*. *sphaeroides* (PDB: 4FHZ); PfEstII, esterase II from *P*. *fluorescens* (PDB: 1AUO); PA3859, from *P*. *aeruginosa* (PDB: 3CN9); FTT258, from *F*. *tularensis* (PDB: 4F21); APT1, from human (PDB: 1FJ2); LYPLAL1, from human (PDB: 3U0V). Identical and similar residues among groups are shown in white font on a red background and in red font on a white background, respectively. Triangles represent the locations of the catalytic active sites (serine (S), aspartate (D) and histidine (H)) and squares represent the residues located on the oxyanion hole (tyrosine (Y)/tryptophan (W)/valine (V)/leucine (L)/serine (S) and glutamine (Q)/methionine (M)). Black boxes represent the locations of residues Arg79 and Arg83 of PE8. The secondary structural elements α-helices, 3_10_-helices and β-strands of PE8 are denoted by α, η and β, respectively, with symbols above the sequences.

**Figure 3:**
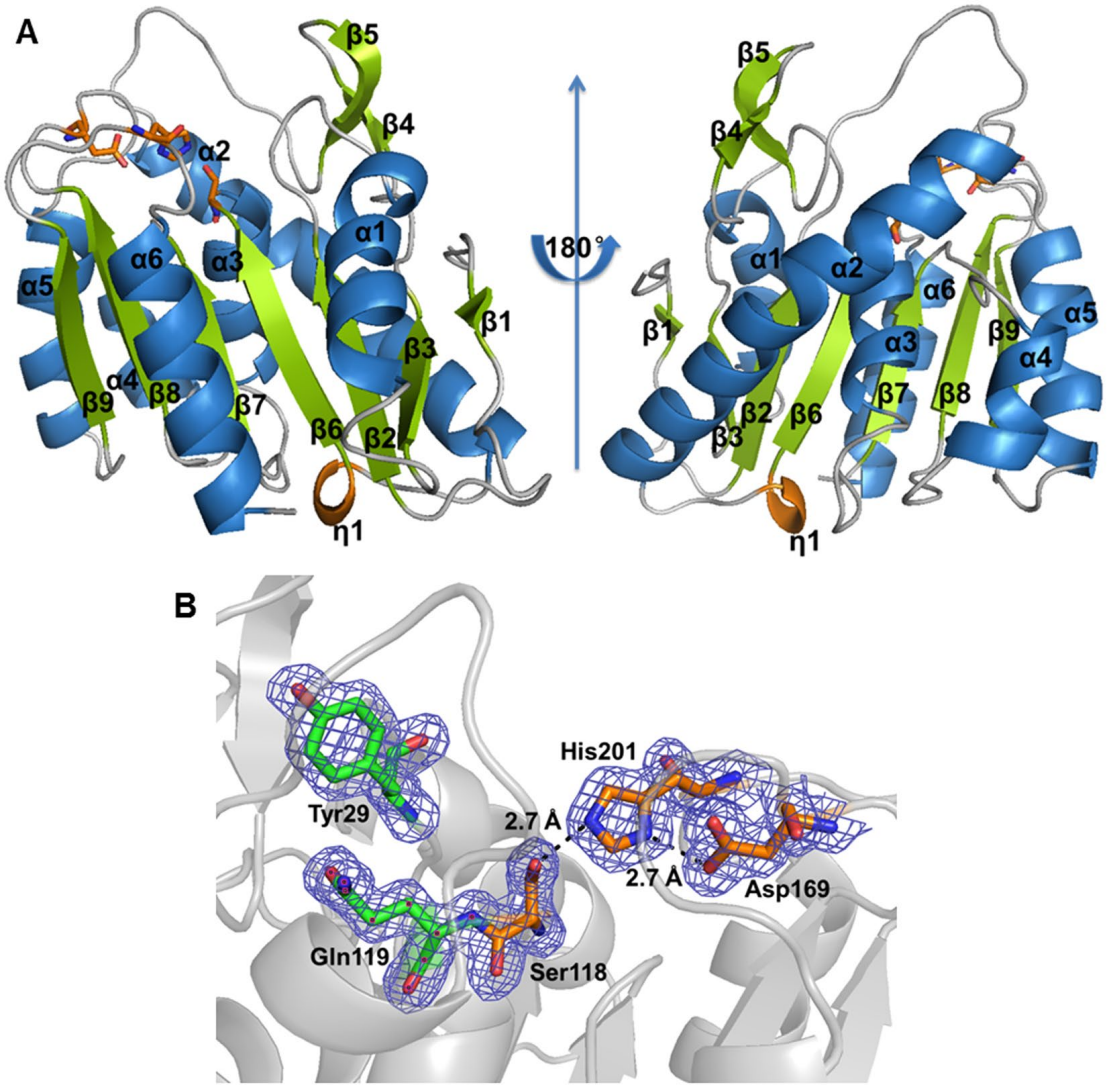
Schematic representation of PE8 structure. (**A**) Cartoon representation of PE8. The α-helices, β-strands, and 3_10_-helices are shown in blue, green, and brown, respectively. The catalytic triad residues are indicated as stick models in orange. (**B**) Visualization of the PE8 active site. The residues of the catalytic triad and oxyanion hole are shown as stick models in orange and green, respectively. Residue His201 forms hydrogen bonds with Ser118 and Asp169. The electronic map is contoured to 1.0 σ at the 2*F*_*o*_ − *F*_*c*_ map.

The molecular structure of PE8 had a typical α/β–hydrolase fold^21–23^, containing seven predominantly parallel β strands (β1, β2, and β5–β9) surrounded by six α helices (α1–α6) (Figs 2 and S1). The β-strands formed a parallel β-sheet in the order of β1 (antiparallel to all the others), β3, β2, β6, β7, β8 and β9, with helices α1 and α6 on one side and α2, α3, α4 and α5 on the other side (Fig. 3A).

Compared with the known structures of other LPCE family members, the main differences were found in the short β-strands, helix α4, the short α helices and the 3_10_ helices (Supplementary Figure S1). Helix α4 connects β7 with β8 in PE8, *R*. *sphaeroides* RspE^12^ and human LYPLAL1^7^. However, in *P*. *fluorescens* esterase II^14^, *P*. *aeruginosa* PA3859^13^ and human APT1^15^, strands β7 and β8 are connected by long loops containing short α helices or 3_10_ helices. In addition, on the loop between strand β3 and helix α2 (i.e., loop β3), there are four short antiparallel β-strands (β4, β5, βA and βB) in esterase II and APT1. In contrast, βA and βB of loop β3 are replaced with a short helix in RspE or a winding loop in PE8, PA3859 and LYPLAL1 (Supplementary Figure S1).

The β-strands β6, β8 and β9 provide the framework onto which the catalytic residues (Ser118, Asp169 and His201 in PE8) are placed (Figs 2 and 3A). The remaining β strands and α helices are not directly involved in the formation of the catalytic site, and thus, the differences in the secondary structure mentioned above may not influence the catalysis of the active site directly.

### Active site

Sequence analysis and the three-dimensional (3D) structure revealed that the catalytic triad residues of PE8 consist of Ser118, Asp169 and His201, which are located on the C-terminal sides of β strands β6, β8 and β9 in the central β sheets (Figs 2 and 3) and are conserved in esterases^1^. The catalytic site is located on the loops outside of the α/β/α-sandwich structure, and no lid covers the catalytic site. The catalytic residue Ser118 is located in the conserved GFSQG motif (Fig. 2). The hydrogen bond distance within the catalytic triad is 2.7 Å from Ser118-Oγ to His201-Nε2 and 2.7 Å from His201-Nδ1 to Asp169-Oδ1 (Fig. 3B). To confirm the roles of the three amino acid residues, site-directed mutagenesis was performed to replace these residues with alanine. The activities of mutant S118A against *p*-nitrophenyl (*p*-NP) acetate, *p*-NP butyrate, *p*-NP hexanoate and *p*-NP octanoate were 19.5 ± 0.2%, 16.3 ± 0.5%, 1.3 ± 0.2% and 0%, respectively, compared with the wild-type enzyme (Fig. 1C). The replacement of catalytic residues Ser118 or Asp169 with alanine led to a complete loss of activity against *p*-NP acetate, *p*-NP hexanoate and *p*-NP octanoate, with approximately only 5% of residual activity retained against *p*-NP butyrate (Fig. 1C).

Compared with the known structures of LPCE family homologs, the oxyanion hole of PE8 is likely formed by nitrogen atoms of Tyr29 and Gln119, as observed for RspE (Tyr and Gln) but not for esterase II, PA3859, FTT258 and human APT1 (Leu and Gln) or human LYPLAL1 (Ser and Gln) (Figs 2 and 3B). In PE8, the oxyanion hole is occupied by a water molecule in each chain (data not shown). These water molecules might be candidates for the nucleophilic attack on the acylated enzyme^13^, followed by the release of the enzyme in its resting form.

### Structural comparison of PE8 with other LPCE family homologs

**Figure 4:**
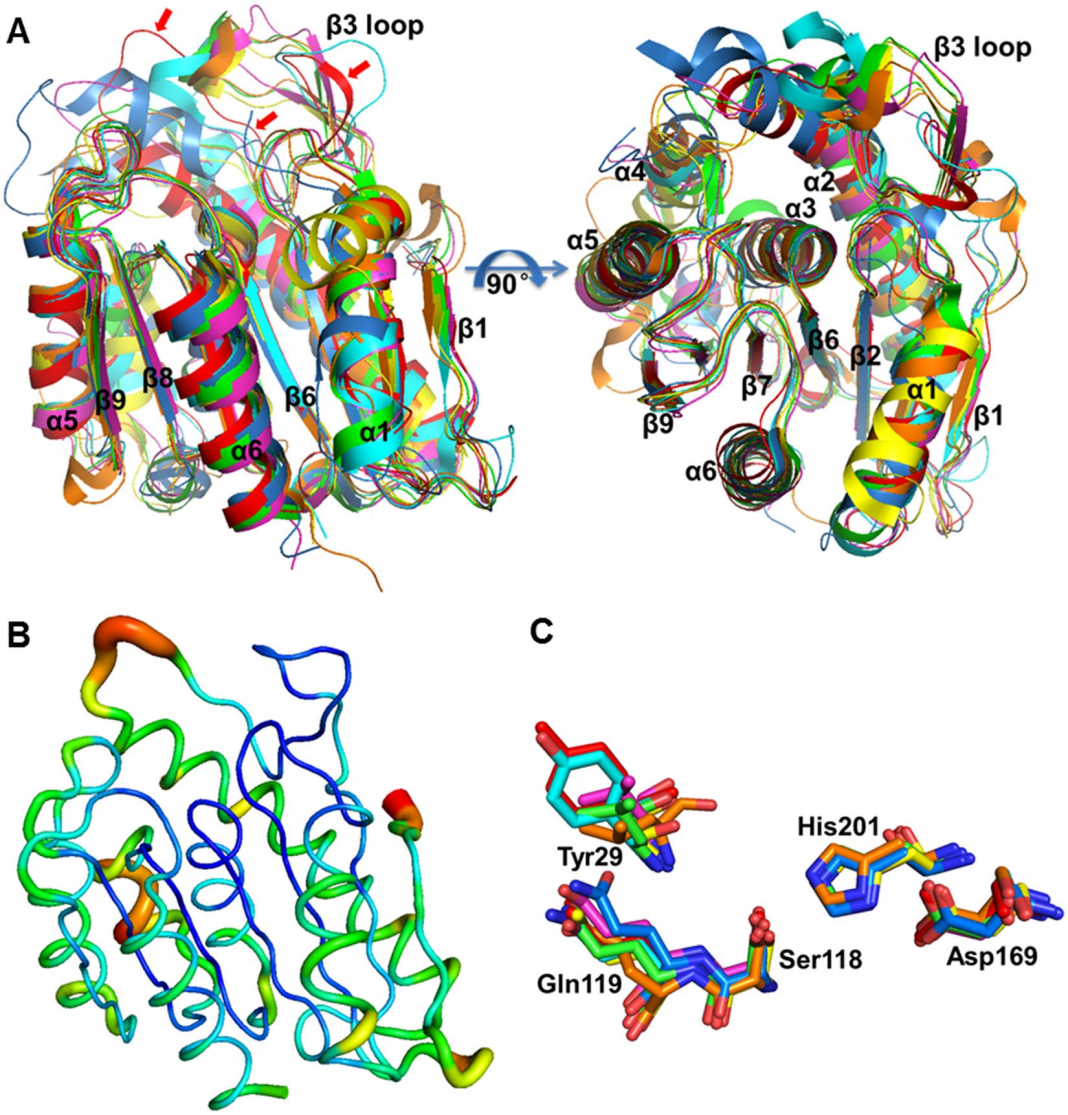
A structural comparison of PE8 with other homologous esterases. (**A**) The structural superposition of PE8 (red, PDB: 5DWD), *R*. *sphaeroides* RspE (cyan, PDB: 4FHZ), *P*. *fluorescens* esterase II (magentas, PDB: 1AUO), *P*. *aeruginosa* PA3859 (green, PDB: 3CN9), human APT1 (orange, PDB: 1FJ2), LYPLAL1 (yellow, PDB: 3U0V) and *F*. *tularensis* FTT258 (blue, PDB: 4F21), which was performed in PyMOL using Cα atoms with default parameters. The β-strands, α-helices and winding β3 loops are labeled. Red arrows indicate the β3 loop of PE8. (**B**) The B-factor distribution of PE8. Wider and redder tubing corresponds to higher B-factors. (**C**) The comparison of the catalytic triads and oxyanion holes among the seven homologous proteins. The five residues are presented as stick models. The colors have the same meaning as in (**A**).

A search for related models in the Protein Data Bank (PDB) using DALI^24^ yielded the best match with homologs belonging to the LPCE family, giving alignments of 197–212 residues (22–46% identity) with root-mean-square deviations (RSMD) of 2.4–1.7 Å (Table 2). The homolog with the highest structural similarity and the highest sequence identity was *R*. *sphaeroides* RspE (PDB ID 4FHZ, Z score = 33.9, RMSD = 1.7 Å for 212 Cα atoms, identity = 46%, Table 2), followed by esterase II (PDB 1AUO), PA3859 (PDB 3CN9), APT1 (PDB 1FJ2), LYPLAL1 (PDB 3U0V) and FTT258 (PDB 4F21). Although the sequence identities between PE8 and the five homologs are relatively low, ranging from 22% to 26%, these homologs still show high 3D structural similarities with low RSMD (2.1–1.7 Å), mainly because of their relatively conserved α/β–hydrolase fold, which contains a β-sheet with seven strands surrounded by five or six α helices (Fig. 4A).

**Table 2:** Structural homologs of PE8 as revealed by DALI^22^.

Esterase	PDB ID	Z score	RSMD (Å)	NALI^a^	NRES^b^	Identity (%)
*R*. *sphaeroides* RspE	4FHZ	33.9	1.7	212	220	46
*P*. *fluorescens* esterase II	1AUO	28.0	2.1	208	218	24
*P*. *aeruginosa* PA3859	3CN9	27.8	2.4	207	214	26
Human APT1	1FJ2	27.0	2.3	208	229	23
Human LYPLAL1	3U0V	25.4	2.4	203	222	22
*F*. *tularensis* FTT258	4F21	24.6	2.1	197	220	22

^a^NALI: number of aligned residues; ^b^NRES: total number of residues.

Superimposing these homologs also revealed their similar features and overall folds. The core α/β–hydrolase fold structure, including the catalytic triad and oxyanion hole, is highly conserved, especially in β strands β6, β8 and β9 and helices α3, α5 and α6 in the loops (Fig. 4A,C). High structural variability can be observed in helices α1, α4, and the β3 loop (Fig. 4A), suggesting that these structures are not essential for the catalytic activity.

Among these regions, the β3 loop shows the most significant variation in its amino acid sequence and secondary and 3D structures (Figs 2,4 and S1). The β3 loop shows a higher B-factor within the PE8 structure (Fig. 4B), implying flexibility in its structure and function. Highly variable loops have also been described in LPCE family homologs previously^11, 15^. This winding loop is believed to be responsible for the substrate specificity and conformational changes of these homologs^13, 25^ and is observed to cause the open and closed conformations and affect the catalytic activity and membrane binding of *F*. *tularensis* FTT258^11^.

### A new substrate-binding pattern within the LPCE family

**Figure 5:**
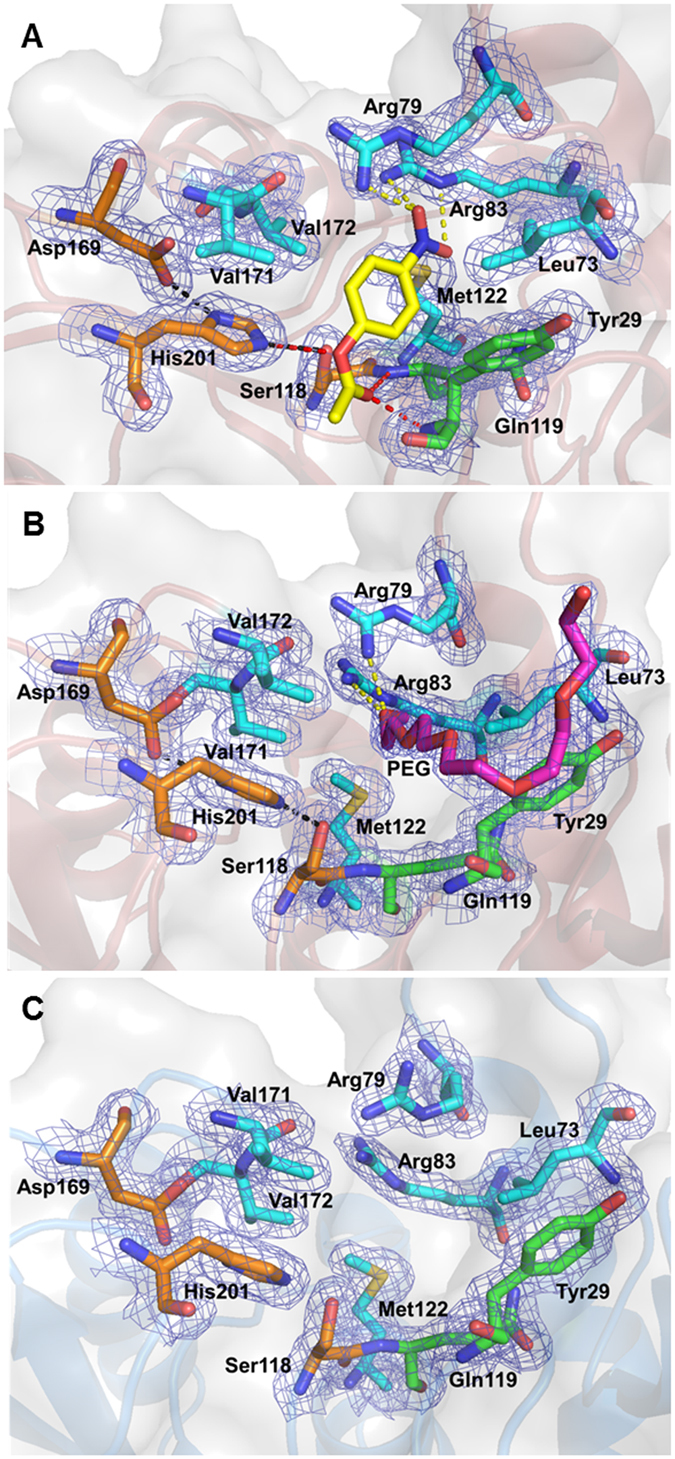
Visualization of the substrate-binding site of PE8. (**A**) The structural model of PE8 and its complex with substrate *p*-NP acetate (yellow sticks). (**B**) The PEG MME 550 molecule in the substrate-binding site of PE8 chain B. (**C**) The substrate-binding site of PE8 chain A (cartoon in blue), showing the different configuration of Arg79 relative to chain A. All the electronic maps are contoured to 1.0 σ at the 2*F*_*o*_−*F*_*c*_ map. The residues Leu73, Arg79, Arg83, Met122, Val171 and Val172, the catalytic triad and the oxyanion hole of PE8 are shown as stick models. Residue His201 forms hydrogen bonds with Ser118 and Asp169 (black dotted lines). The substrate molecule or PEG MME 550 forms hydrogen bonds with His201, Tyr29 and Gln119 (red dotted lines) and Arg79 and Arg83 (yellow dotted lines).

PE8 exhibited maximum activity toward the substrate *p*-NP acetate^18^. A docking study of *p*-NP acetate was performed to explore the interaction between PE8 and the substrate. The substrate successfully docked into the active site of PE8 (Fig. 5A), where a PEG MME 550 molecule was detected in the crystal structure (Fig. 5B). In the enzyme-substrate complex docking model, the alcohol part of the substrate occupied the hydrophobic substrate-binding pocket, which was formed by hydrophobic residues Leu73, Met122, Val171 and Val172 (Fig. 5A), similar to the previously suggested mechanism in the LPCE family^11^. The distance between the residues and the nearest carbon atom of the alcohol part of the substrate was 3.3–4.1 Å. More importantly, we found that the two nitro-O atoms of *p*-NP acetate might form one and two hydrogen bonds with the side chain N atoms of Arg79 and Arg83, respectively (Fig. 5A). To the best of our knowledge, this hydrogen bond between the alcohol part of the substrate and the binding site of the esterase (i.e., not the catalytic site) has not been reported before^11–14^.

**Figure 6:**
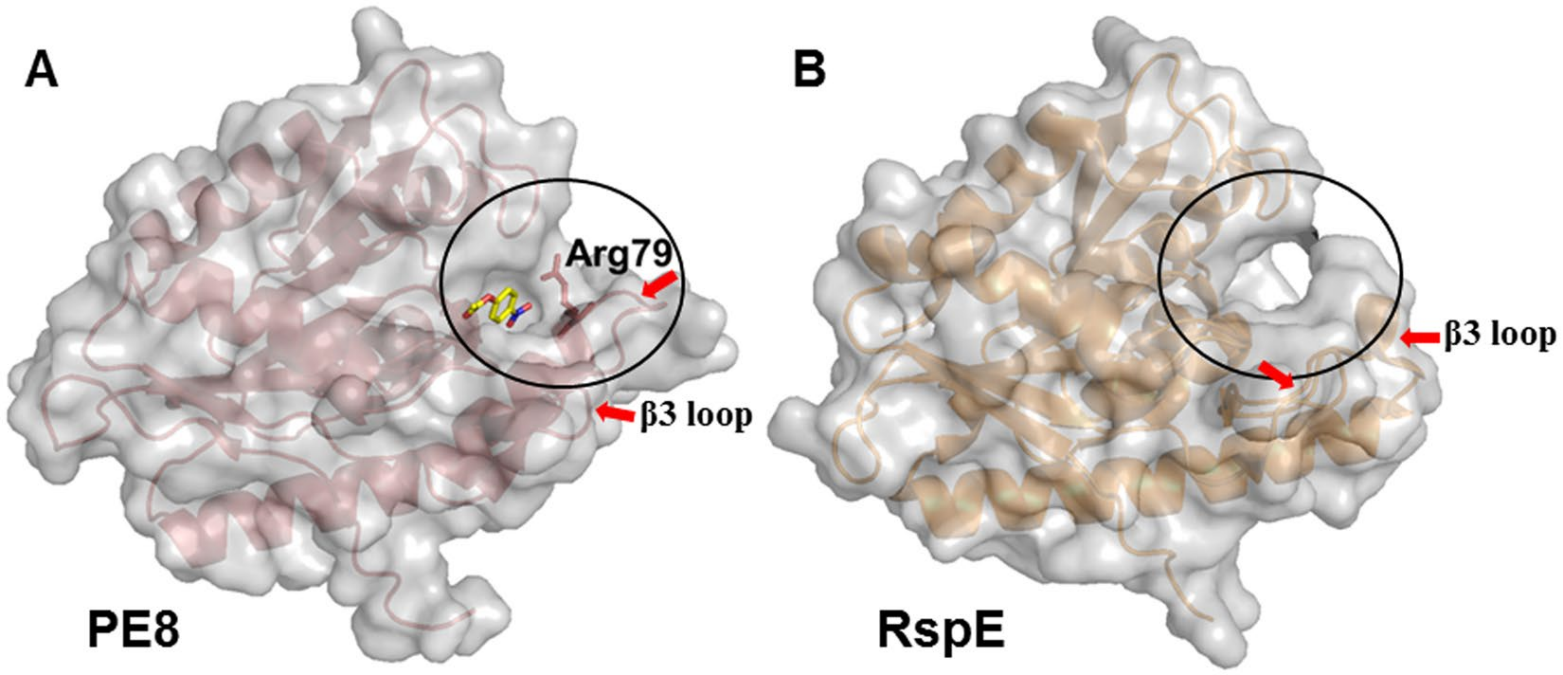
Molecular surface representation (light grey) of PE8 and *R*. *sphaeroides* RspE (PDB 4FHZ). (**A**) *p*-NP acetate docks into the substrate-binding pocket of PE8 chain B, which is shown as a cartoon representation in red (Arg79 is shown as a stick). (**B**) RspE is shown as a cartoon representation in orange. The relatively closed cavity of the substrate-binding pocket caused by Arg79 in PE8 and the open cleft in RspE are indicated with black circles. Red arrows indicate the β3 loops.

In addition, two different configurations of the side chain of Arg79 were observed in chain A and chain B of PE8 (Fig. 5B and C). The configuration of Arg79 in chain B was proposed as the substrate-binding configuration because it was occupied by the PEG molecule, whereas that in chain A might correspond to the releasing state of the substrate-binding site. Thus, the substrate might be bound and subsequently released by the configuration switching of Arg79. Arg79 is located on the β3 loop of the structure of PE8. It forms a closed wall between the long winding β3 loop and the loop between strand β8 and helix α5, in which the Asp169, Val171 and Val172 are located (Figs 2 and 6A). This wall makes the alcohol binding pocket of PE8 a relatively closed cavity and forms stronger interactions with the alcohol group of the ester substrate. However, because of the high sequence variability and structural flexibility of loop β3 (Figs 2 and 4), no similar structure was found in other homologs of the LPCE family. For example, *R*. *sphaeroides* RspE has the highest sequence and structural similarity with PE8; however, the substrate-binding pocket of *R*. *sphaeroides* RspE is an open, longer cleft (Fig. 6B), similar to other members of the LPCE family^11, 13^. Hence, Arg79 may play an important role in substrate binding and the shape of the binding pocket, thereby conferring substrate specificity to PE8.

To investigate the relationship between the supposed substrate-binding-related residues and the hydrolysis activity of PE8, mutants L73A, R79A, R83A, M122A, V171A and V172A were constructed by site-directed mutagenesis, and their catalytic activities against *p*-NP esters and kinetic parameters for the hydrolysis of *p*-NP acetate were determined (Fig. 1C and Table 3). The *K*m, *k*cat and *k*cat/*K*m of wild-type PE8 were 0.66 ± 0.049 mM, 30 ± 0.68 s^−1^ and 45 mM^−1^ s^−1^, respectively. The catalytic activities and *K*m and *k*cat values of L73A, R83A and V172A were similar to those of wild-type PE8, indicating that these mutations had little effect on the catalytic activity and substrate affinity of PE8 using *p*-NP esters. A slight increase in the *K*m value and decrease in the *k*cat value of mutant V171A were observed (0.89 ± 0.070 mM and 19 ± 0.42 s^−1^, respectively), suggesting that the ability to bind the substrate was weakened and that the turnover rate of the enzyme-substrate complex to the product and enzyme was decreased. Thus, the catalytic efficiency against *p*-NP acetate of mutant V171A was reduced by nearly half, as indicated by a *k*cat/*K*m value of 21 mM^−1^ s^−1^. The *k*cat and *k*cat/*K*m values of mutant R79A were approximately 2-fold higher than those of wild-type PE8. Considering the structure of Arg79 shown above, replacing arginine with alanine probably removed the barrier and expanded the substrate-binding pocket, thereby accelerating substrate access and exit. Interestingly, mutant M122A showed a large decrease in its *K*m value (0.075 ± 0.0069 mM) toward *p*-NP acetate, resulting in a 10-fold improvement of *k*cat/*K*m (553 mM^−1^ s^−1^). In addition, the catalytic activities of mutant M122A against *p*-NP butyrate, *p*-NP hexanoate and *p*-NP octanoate also increased relative to those of wild-type PE8 (Fig. 1C). The substitution of methionine by alanine might cause less steric repulsion of the substrates and remove the barrier to substrate access. Hence, further engineering in positions Arg79, Met122 or Val171 may provide high activity, affinity or selectivity mutants to specific substrates to facilitate the development of structure-based therapeutics and other biocatalytic applications.

**Table 3:** Kinetic parameters of PE8 and its mutants.

Enzyme	*V*max (μM/min/mg)	*K*m (mM)	*k*cat (s^−1^)	*k*cat/*K*m (mM^−1^ s^−1^)^a^
PE8	71 ± 1.6	0.66 ± 0.049	30 ± 0.68	45 (100%)
L73A	55 ± 0.64	0.53 ± 0.026	23 ± 0.27	43 (96%)
R79A	132 ± 3.4	0.60 ± 0.062	55 ± 1.7	92 (204%)
R83A	97 ± 1.0	0.60 ± 0.023	41 ± 0.42	68 (151%)
M122A	96 ± 1.6	0.075 ± 0.0069	40 ± 0.68	533 (1184%)
V171A	36 ± 1.8	0.89 ± 0.070	19 ± 0.42	21 (47%)
V172A	82 ± 1.5	0.50 ± 0.035	35 ± 0.63	70 (156%)
S118A	12 ± 0.32	0.54 ± 0.046	5.1 ± 0.14	9.4 (21%)

^a^Percentages in parentheses were calculated relative to PE8. All catalytic reactions were performed in triplicate in 100 mM Tris-HCl buffer (pH 7.5) buffer at 30 °C using *p*-nitrophenyl acetate as the substrate at concentrations of 0.05–4 mM.

## Conclusion

In this study, a new esterase structure in the LPCE family is presented. The structural information of *P*. *halotolerans* PE8 expands our knowledge of the catalytic mechanisms of the LPCE family and provides new insight into the substrate-binding pattern in this family. The results establish a novel approach for developing specific inhibitors of its homologs, which could be used for mechanistic research and targeted therapy. In addition, the results of this paper may help broaden the applications of the LPCE family members as biocatalysts in industry.

## Materials and Methods

### Sequence analysis

The PE8 coding gene was identified and cloned from the genome of *P*. *halotolerans* B2^T^ ^16, 18^. Amino acid sequence analysis was conducted by BLASTp against the PDB from the National Center for Biotechnology Information (NCBI). Multiple sequence alignment of homologs belonging to the LPCE family was performed by ClustalX v. 2^26^. Secondary structure assignment was determined by DSSP v. 2.0^27^ and PROMOTIF^28^. The alignment result with the secondary structure was visualized using ESPript 3.0^29^.

### Mutation, protein expression and purification

Point mutants were generated by site-directed mutagenesis using wild-type plasmid as a template for the polymerase chain reaction (PCR). A 15-cycle reaction was performed with the following steps: 98 °C for 10 sec, 55 °C for 30 sec, and 72 °C for 3 min per cycle with PrimeSTAR HS DNA polymerase (Takara, Dalian, Liaoning, China). After digestion with enzyme *Dpn*I (New England Biolabs, Beverly, MA, USA), the PCR products were transformed into *Escherichia coli* DH5α cells. The positive constructs were determined by DNA sequencing. The wild-type and mutant forms of PE8 were cloned into the expression vector pET28b (Novagen, Madison, WI, USA) and were expressed in *E*. *coli* Rosetta (DE3) cells induced by 0.5 mM isopropyl-β-D-thiogalactopyranoside (IPTG) for 16 hours at 20 °C, as described previously^18, 30^. Cells were harvested and disrupted by a sonicator or high-pressure homogenizer. The lysates were sequentially purified by Ni-NTA affinity and size-exclusion chromatographys (SEC). The Superdex-200 column was calibrated with protein size markers: thyroglobulin (670 kDa), gamma globulin (158 kDa), ovalbumin (44 kDa), myoglobin (17 kDa) and vitamin B12 (1.35 kDa). The protein concentration was determined by the Bradford method.

### MALS analysis

MALS analysis was performed to estimate the MW of PE8 in the National Center for Protein Science Shanghai (NCPSS). First, 20 μl of 1.5 mg/ml purified PE8 protein was subjected to SEC-MALS using a WTC-030S5 size-exclusion column (Wyatt, Santa Barbara, CA, USA) with elution buffer (20 mM Tris-HCl, pH 7.4, 100 mM NaCl) and passed in tandem through a Wyatt DAWN HELEOS II light scattering instrument (Wyatt) and an Optilab rEX refractometer (Wyatt). Data collection and analysis were performed with Astra 6 software (Wyatt).

### Biochemical characterization of PE8 and its mutants

The standard reaction was carried out with the appropriate amount of purified PE8 or its mutants in 1 ml mixtures containing 100 mM Tris-HCl (pH 7.5) buffer and 1 mM *p*-NP acetate (Sigma-Aldrich, Milwaukee, WI, USA, dissolved in acetonitrile)^18^. The activities were determined at 30 °C and 405 nm using a DU800 UV/Visible spectrophotometer (Beckman, Houston, TX, USA). All experiments were performed in triplicate and corrected for substrate autohydrolysis. Substrate specificity assays were performed with *p*-NP acetate, *p*-NP butyrate (Sigma-Aldrich), *p*-NP hexanoate (TCI, Tokyo, Japan) and *p*-NP octanoate (Sigma-Aldrich).

The kinetic parameters were obtained using *p*-NP acetate as a substrate at different concentrations (0.05 to 4 mM). The kinetic parameters were calculated by analyzing the slopes of the Michaelis-Menten equation using GraphPad Software (GraphPad Inc., USA).

### Crystallization, data collection, and structure determination

Crystals of PE8 protein were obtained using the “hanging drop” method by mixing 1 µl of 20 mg/ml protein with 1 µl of reservoir solution at 20 °C. The reservoir buffer contained 0.05 M CaCl_2_, 0.1 M Bis-Tris, and 25% (v/v) PEG MME 550 (pH 6.5). The X-ray diffraction datasets were integrated, scaled and merged using the HKL3000 program^31^. Phases were obtained by molecular replacement using Phaser^32^ with the PDB coordinates 4FHZ (*R*. *sphaeroides* RspE)^14^ as the initial model. The refinement was conducted by Refmac5^33^ in the CCP4 software suite^34^ and Phenix^35^. The model was built manually by Coot^36^. A structural similarity search was performed with the DALI server^24^. Docking studies were performed with AutoDockTools program^37^. The ligands for docking were edited by Avogadro software^38^, and the topologies of the ligands were generated using the PRODRG server^39^. The successful docking conformation should be satisfied the following criteria: the distance between the OG atom of serine and the carbonyl carbon atom of the substrate was about 2 Å; the catalytic hydrogen bonds were formed, including that between Hδ of the catalytic histidine and the ester oxygen of the substrate, as well as those between the carbonyl oxygen of the substrate and the nitrogen atoms of oxyanion hole residues (Tyr29 and Gln119)^40, 41^. All the structures were drawn using PyMOL software (http://pymol.sourceforge.net).

The structure of PE8 was deposited in PDB with accession number 5DWD.

## Electronic supplementary material

Supplementary Information
