# Supplementary material for: Crystal structure of Pelagibacterium halotolerans PE8: New insight into its substrate-binding pattern
Source: Sci Rep. 2017 Jun 30;7:4422. doi: 10.1038/s41598-017-04550-7 (PMC5493697; doi:10.1038/s41598-017-04550-7)

Crystal structure of *Pelagibacterium halotolerans* PE8: New insight into its substrate-binding pattern

Ying-Yi Huo[1], Suhua Li[2], Jing Huang[2], Zhen Rong[1], Zhao Wang[3], Zhengyang Li[2], Rui Ji[2], Siyun Kuang[2], Heng-Lin Cui[3], Jixi Li[2]*, Xue-Wei Xu[1]*

[1] Key Laboratory of Marine Ecosystem and Biogeochemistry, Second Institute of Oceanography, State Oceanic Administration, Hangzhou 310012, China

[2] State Key Laboratory of Genetic Engineering, Collaborative Innovation Center of Genetics and Development, School of Life Sciences, Shanghai Engineering Research Center of Industrial Microorganisms, Fudan University, Shanghai 200438, China

[3] College of Food and Biological Engineering, Jiangsu University, Zhenjiang 212013, China

*Co-correspondence: xuxw@sio.org.cn or lijixi@fudan.edu.cn

Supporting Information

Supplementary Figure S1. Topology diagram of homologs from the LPCE family. (A) PE8 (PDB 5DWD), (B) *R. sphaeroides* RspE (PDB 4FHZ), (C) *P. fluorescens* esterase II (PDB 1AUO), (D) *P. aeruginosa* PA3859 (PDB 3CN9), (E) human LYPLAL1 (PDB 3U0V), (F) human APT1 (PDB 1FJ2). Violet arrows, β-strands; green rods, α-helices; brown rods, 3_{10}-helices.

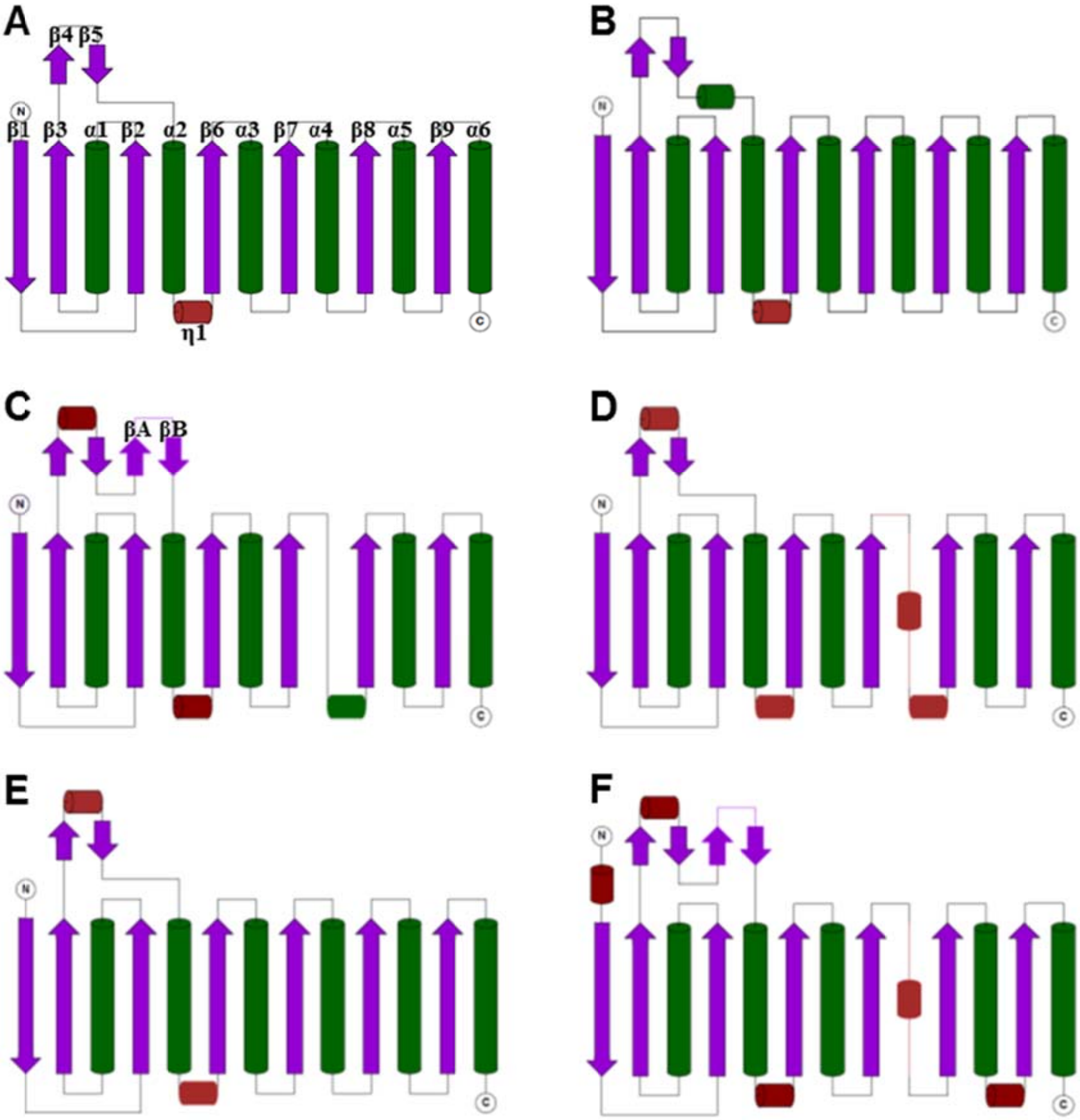

Supplement: Supplementary file 1 — Supplementary Information [file 41598_2017_4550_MOESM1_ESM.pdf]
